# Community-level educational attainment and dementia: a 6-year longitudinal multilevel study in Japan

**DOI:** 10.1186/s12877-021-02615-x

**Published:** 2021-11-23

**Authors:** Tomo Takasugi, Taishi Tsuji, Masamichi Hanazato, Yasuhiro Miyaguni, Toshiyuki Ojima, Katsunori Kondo

**Affiliations:** 1 Department of Community Health and Preventive Medicine, Hamamatsu University School of Medicine, 1-20-1 Handayama, Higashi-ku, Hamamatsu, Shizuoka, 431-3192 Japan; 2 Faculty of Health and Sport Sciences, University of Tsukuba, 3-29-1 Otsuka, Bunkyo-Ward, Tokyo, 112-0012 Japan; 3 Center for Preventive Medical Sciences, Chiba University, 1-33 Yayoi-cho, Inage-Ward, Chiba City, Chiba 263-8522 Japan; 4 Faculty of Social Welfare, Nihon Fukushi University, Okuda, Mihama-cho, Chita-gun, Aichi 470-3295 Japan; 5 Center for Gerontology and Social Science, National Center for Geriatrics and Gerontology, 7-430 Morioka-cho, Obu-City, Aichi 474-8511 Japan

**Keywords:** Socioeconomic status, Education, Cognitive decline, JAGES cohort, Multilevel analysis

## Abstract

**Background:**

As the understanding of the association between community-level education and dementia is insufficient, this study examined the contextual association of community-level prevalence of low educational attainment on the risk of dementia incidence. With this study, we further explored the potential differences in the aforementioned associations for urban and non-urban areas.

**Methods:**

We analyzed 6 years of prospective cohort data from the Japan Gerontological Evaluation Study, beginning with the baseline data collected between 2010 and 2012, for 51,186 physically and cognitively independent individuals aged ≥65 years (23,785 men and 27,401 women) from 346 communities in 16 municipalities across 7 prefectures. We assessed dementia incidence using available data from the long-term care insurance system in Japan. We dichotomized education years as ≤9 and ≥ 10 years and aggregated individual-level educational attainment as a community-level independent variable. Model 1 covariates were age and sex. Income, residential years, disease, alcohol, smoking, social isolation, and population density were added in Model 2. We conducted multiple imputation to address the missing data. We performed a two-level (community and individual) survival analysis to calculate hazard ratios (HRs) and 95% confidence intervals (CIs).

**Results:**

The results indicate that the cumulative incidence of dementia during the follow-up period was 10.6%. The mean proportion with educational attainment of ≤9 years was 40.8% (range: 5.1–87.3%). Low community-level educational attainment was significantly associated with higher dementia incidence (HR: 1.04; 95% CI: 1.01–1.07), estimated by 10 percentage points of low educational attainment after adjusting for individual-level educational years and covariates. While the association was significant in non-urban areas (HR: 1.07; 1.02–1.13), there was no association in urban areas (HR: 1.03; 0.99–1.06).

**Conclusions:**

Older people living in communities with low educational attainment among their age demographic develop dementia more often compared with those living in areas with high educational attainment after adjusting for individual-level educational attainment and covariates; the association was pronounced in non-urban areas. Securing education for adolescents as a life course and population approach could thus be crucial in preventing dementia later in life among older people living in non-urban areas.

**Supplementary Information:**

The online version contains supplementary material available at 10.1186/s12877-021-02615-x.

## Background

Dementia is a major global health issue caused by rapidly aging populations worldwide, with which over 50 million people are currently affected. The total number of people with dementia is expected to reach 82 million by 2030 and 152 million by 2050 [1]. Further, half of the 10 million new dementia cases occur annually in Asia [2]. Dementia not only affects individuals’ personal and family lives and careers but also carries enormous medical and social care costs [3].

Although there is no cure for dementia, a recent review reported that 40% of dementia cases are preventable; the authors stated that attention should be paid to the following 12 risk factors: low education in early life, hearing loss, traumatic brain injury, hypertension, alcohol consumption, obesity in midlife, smoking, depression, social isolation, physical inactivity, air pollution, and diabetes in late life; the second-highest percentage of these risk factors is less education [4]. A systematic review showed that low education is associated with increased cognitive decline or dementia [5]. Recent studies have begun emphasizing the association between community-level education, rather than the individual level, and dementia and cognitive impairment. For example, it is possible to examine how community-level education inequality affects individual-level dementia incidence after adjusting for individual-level education. A cross-sectional study found that low community-level education is associated with declining cognition [6]. However, some studies have found associations between area of residence and dementia, such as a reduction in cognitive decline among adults living in urban areas compared with rural areas [7, 8]. Meanwhile, a recent U.S. study found significantly lower dementia prevalence among adults living in rural areas compared with urban areas over the past few decades, which can be attributed to improvements in educational attainment [9]. In short, explorations of the relationship between community-level educational attainment and dementia among urban and rural older adults have been insufficient [9, 10].

The aim of this longitudinal study using a large-sample data set was to examine the risk of dementia among older populations living in communities with higher proportions of low educational attainment, defined as ≤9 years, compared with the risk in communities with lower proportions of low education attainment. We performed multilevel (community and individual) analyses to investigate the contextual associations of community-level educational attainment on developing dementia among older individuals after we adjusted for confounding factors. Additionally, we explored the potential differences in associations between community-level education and dementia for urban and non-urban areas.

## Methods

### Participants

**Fig. 1:**
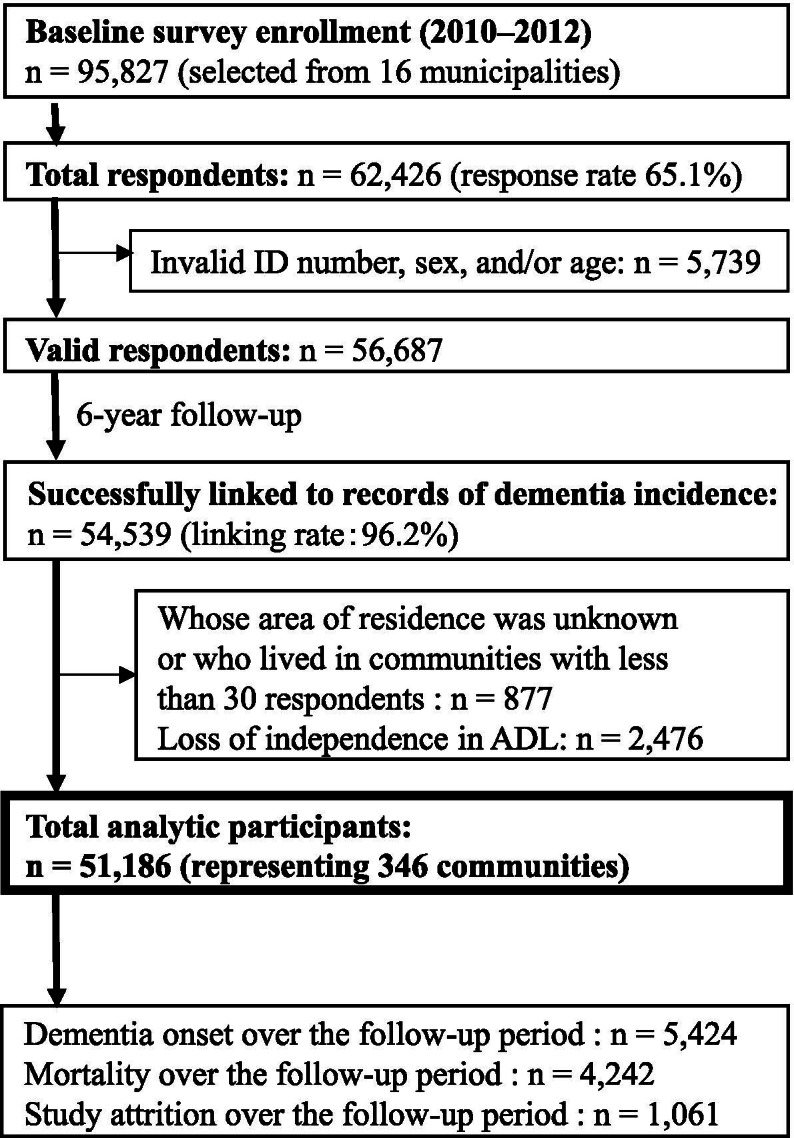
Flow of participants in the cohort study

This research was a 6-year prospective longitudinal study using data from a large sample acquired from the Japan Gerontological Evaluation Study (JAGES). One objective of JAGES is identifying social and behavioral factors associated with dementia onset among physically and cognitively independent individuals aged ≥65 years [11, 12]. For the survey, a baseline mail self-reported questionnaire was administered from August 2010 to January 2012. In the study, 95,827 older people were chosen by random sampling from 16 municipalities in 7 prefectures in Japan, including urban and non-urban areas. Among 62,426 respondents (response rate: 65.1%), 56,687 had valid information in terms of ID number, sex, and age (valid response rate: 59.2%). Of the 56,687 participants with valid responses, 54,539 (96.2%) were successfully linked to the records of dementia incidence during a 6-year follow-up term. A total of 51,186 participants (23,785 men and 27,401 women) were available for the present multilevel survival analyses (Fig. 1). This sample size was determined after excluding 3353 respondents who either lived in communities with fewer than 30 respondents or had limitations in basic activities of daily living, such as walking, bathing, and toileting, to ensure that the sample was both functionally and cognitively independent. Our research protocol and informed consent method were approved by the Human Subjects Committees of Nihon Fukushi University (no. 10–5) and the Chiba University Faculty of Medicine (no. 2493). All methods were carried out in accordance with Declaration of Helsinki.

### Measures

#### Dependent variable

Our outcome variable was dementia incidence based on each municipality’s publicly available long-term care insurance records. Japan’s Ministry of Health, Labor and Welfare (MHLW) established a scale called the Degree of Independency in Daily Lives of Demented Individuals (hereafter “dementia scale”) [13], and each local government sent personal investigators to participants’ homes to assess their eligibility for nursing care, such as need for home helpers. The investigators evaluated physical function, daily life ability, cognitive function, mental and behavioral disorders, adaptation to social life, and special medical treatment within 14 days [14, 15]. Following the assessment, the investigators classified the participants into one of eight ranks on the dementia scale according to their cognitive disability (Supplementary Table S1) [15, 16].

The dementia scale correlated highly with the Mini-mental State Examination (Spearman rank correlation ρ = − 0.74) [17]. Moreover, another study, where neuropsychiatrists conducted clinical interviews as defined by the International Psychogeriatric Association, found that the dementia scale was an effective indicator for the clinical diagnosis. Sensitivity and specificity were 73 and 96% for rank II dementia [18]. According to MHLW, we defined rank II dementia or above as showing some symptoms, behaviors, or communication problems during daily lives [15, 19]. We discontinued data tracking participants who did not develop dementia before they died or were lost to follow-up during the 6-year study period.

### Community- and individual-level independent variables

Educational attainment was measured with the question, “How many years of formal education have you had?” with the following response options: < 6, 6–9, 10–12, and ≥ 13 years. For our study, we dichotomized the variable as ≤9 years or ≥ 10 years [20]. We set the cutoff at 9 years because 9 years of education has been compulsory in Japan since 1947 [21]. We aggregated individual-level educational attainment within the community to be a community-level independent variable.

### Covariates

We evaluated a number of risk factors for dementia as potential confounders in this study that had been identified in earlier research [4, 22]. Age at baseline and sex were distributed based on the municipalities, and we coded age as a continuous variable between 65 and 103. For the community-level covariate, we divided habitable areas’ population density in the participants’ residential school districts into quartiles (≥10,100, 7900–10,099, 3280–7899, or < 3280 persons per square kilometer) [23]. Then, we calculated equivalized household income by dividing household income by the square root of the number of household members, and we grouped annual income into one of the three categories: ≤1.99, 2–3.99, or ≥ 4 million yen [19]. Years of residence were grouped into one of the seven categories: < 5, 5–9, 10–19, 20–29, 30–39, 40–49, or ≥ 50 years. Current medical treatment for diseases known to increase dementia risk (stroke, hypertension, diabetes, and/or hearing loss) [4] was collected with a “yes/no” choice for each disease. For health behaviors, we measured frequency of alcohol consumption (every day, 3–6 days/week, 1–2 days/week, 1–3 days/month, ≤1 day/month) and smoking status (non, past, or current). Further, we asked about the number of household members living with the participants and how frequently the participants met friends and acquaintances. Social isolation has been defined as social contact with other people less than once a month [4], whereas for our study, we defined social isolation as living alone and meeting with others less than once a month [23].

### Classification of urban and non-urban areas

We used the European Union (EU) and Organisation for Economic Co-operation and Development (OECD) definition of a functional urban area (FUA). An FUA combines multiple municipalities and comprises a city and its surroundings with less densely populated local units that are part of the commuting areas, and FUAs are excellent for comparing socioeconomic status (SES) among cities [24]. FUAs are classified into the following four categories: 1) small urban area: population below 200,000; 2) medium-sized urban area: population 200,000–500,000; 3) metropolitan area: population 500,000 to 1.5 million; and 4) large metropolitan area: population above 1.5 million [25]. For this study, we set the cutoff population for an urban area at 500,000 because most economic activity in Japan is concentrated in metropolitan and large metropolitan areas [25]. Therefore, we grouped metropolitan and large metropolitan areas into the category of urban area and categorized all other areas as non-urban [26]. By this division, 5 municipalities were urban areas and 11 were non-urban.

### Statistical analyses

We conducted multiple imputation based on multivariate normal imputation to address the missing data [27]. Specifically, we used 20 imputed data sets for analysis, with inferences for the regression coefficients acquired by merging the results over the imputed data sets applying Rubin’s rules [28]. The imputed model contained dementia onset, educational attainment, income, social isolation, years of residence, disease status, and health behaviors. We performed a multilevel survival mixed-effects parametric analysis incorporating individual- and community-level factors. Because a recent research reported that community-level factors had different impacts on urban and non-urban areas, we modeled the two separately [9]. Then, we calculated the hazard ratio (HR) and the 95% confidence interval (CI) for developing dementia during the follow-up term. The HR of community-level educational attainment was estimated as a 10 percentage point difference in aggregated educational attainment [23]. We included community- and individual-level educational attainment and cross-level interaction terms in the crude model and assessed whether the contextual association of community depended on individual-level attributes. Thereafter, we added individual-level covariates (age and sex) in Model 1 and added the community-level covariate and other individual-level covariates (income, years of residence, stroke, hypertension, diabetes, hearing loss, frequency of drinking, smoking status, and social isolation) in Model 2. We used STATA/MP 17 (Stata Corp., College Station, TX) for all statistical analyses.

## Results

The 51,186 participants in this study contributed 267,383 person-years. The median follow-up period was 2120 days, with an interquartile range of 251. In the study, 5424 participants (10.6%) acquired dementia during the follow-up period, with an incidence per 1000 person-years of 20.3 people. There were 4242 and 1061 fatalities and losses to attrition, respectively (Fig. 1). Table 1 presents all respondents’ demographics and socioeconomic characteristics stratified by urban versus non-urban areas. Overall, most participants in non-urban areas had an educational attainment of ≤9 years and an equivalized income of ≤1.99 million yen. We calculated the mean (SD) and range for proportion of educational attainment of ≤9 years in each community, which was 40.8% (17.1%) and 5.1–87.3%, respectively (Table 2).

**Table 1:** Descriptive Statistics of All Individual-level Variables

	Urban		Non-urban		Total	
***Individual-level variables***	n	%	n	%	n	%
**Total**	30,219	59.0%	20,967	41.0%	51,186	100.0%
**Dementia onset**						
No-dementia	27,239	90.1%	18,523	88.3%	45,762	89.4%
Dementia	2980	9.9%	2444	11.7%	5424	10.6%
**Educational attainment (year)**						
Less than 9	12,865	42.6%	12,394	59.1%	25,259	49.3%
10 or longer	17,320	57.3%	8545	40.8%	25,865	50.5%
Other and data missing	34	0.1%	28	0.1%	62	0.1%
**Sex**						
Male	14,295	47.3%	9490	45.3%	23,785	46.5%
Female	15,924	52.7%	11,477	54.7%	27,401	53.5%
**Age**						
65–74	17,943	59.4%	11,642	55.5%	29,585	57.8%
75–84	10,622	35.2%	7814	37.3%	18,436	36.0%
≧85	1374	4.5%	1791	8.5%	3165	6.2%
**Equivalized income (yen)**						
Less than 1,999,999	14,065	46.5%	11,815	56.3%	25,880	50.6%
2,000,000–3,999,999	12,211	40.4%	7078	33.8%	19,290	37.7%
4,000,000 or higher	3694	12.2%	1869	8.9%	5563	10.9%
Data missing	248	0.8%	205	1.0%	453	0.9%
**Years of residence**						
Less than 5	373	1.2%	477	2.3%	850	1.7%
5–9	430	1.4%	597	2.8%	1028	2.0%
10–19	1074	3.6%	1185	5.7%	2259	4.4%
20–29	1463	4.8%	1165	5.6%	2629	5.1%
30–39	2797	9.3%	1823	8.7%	4620	9.0%
40–49	5745	19.0%	2986	14.2%	8731	17.1%
50 or longer	18,315	60.6%	12,712	60.6%	31,027	60.6%
Data missing	21	0.1%	21	0.1%	43	0.1%
**Social isolation**						
No	29,317	97.0%	20,503	97.8%	49,820	97.3%
Yes	852	2.8%	429	2.0%	1280	2.5%
Data missing	50	0.2%	35	0.2%	85	0.2%
**Disease status in treatment**						
Stroke: Yes	331	1.1%	291	1.4%	622	1.2%
Hypertension: Yes	11,965	39.6%	8553	40.8%	20,518	40.1%
Diabetes: Yes	5132	17.0%	3364	16.0%	8496	16.6%
Hearing loss: Yes	1986	6.6%	1679	8.0%	3665	7.2%
**Frequency of drinking**						
Every day	8824	29.2%	5861	28.0%	14,685	28.7%
3–6 days/week	10,917	36.1%	7605	36.3%	18,522	36.2%
1–2 days/week	5017	16.6%	3547	16.9%	8565	16.7%
1–3 days/month	3039	10.1%	2112	10.1%	5151	10.1%
Less than 1 day/month	1540	5.1%	1188	5.7%	2728	5.3%
Data missing	881	2.9%	654	3.1%	1536	3.0%
**Smoking status**						
Non	17,236	57.0%	12,751	60.8%	29,987	58.6%
Past	9612	31.8%	6003	28.6%	15,615	30.5%
Current	3235	10.7%	2101	10.0%	5336	10.4%
Data missing	136	0.4%	113	0.5%	248	0.5%

**Table 2:** Descriptive Statistics of Community-level Variables

	Urban		Non-urban		Total	
***Community-level Variables***	n	%	n	%	n	%
**Total**	292	84.4%	54	15.6%	346	100.0%
**Proportion of Educational attainment**						
Mean (SD)		40.8%	(17.1%)			
(Min.–Max.)		(5.1–87.3%)				
**Population density (persons per square km of inhabitable area)**						
Highest quartile (≥10,100)		86				
Second quartile (7900–10,099)		87				
Third quartile (3280–7899)		86				
Lowest quartile (< 3280)		87				

Table 3 shows the results of the multilevel survival analyses for incident dementia. As per the analysis results for all participants, Model 2—including the community-level covariate and individual-level covariates as well as community-level high prevalence of low educational attainment—demonstrated a significant relationship with dementia risk (HR: 1.04; 95% CI: 1.01–1.07), estimated by 10 percentage points of increment of a proportion of educational attainment in a community area. Individual-level educational attainment showed a significant association with dementia probability (HR: 1.08; 95% CI: 1.01–1.16 in Model 2). However, there were no significant cross-level interaction terms in Models 1 and 2.

**Table 3:** Multilevel Survival Analysis for Developing Dementia Onset (Participants Nested in 346 Community Areas)

	Crude			Model 1			Model 2		
	HR	95% CI		HR	95% CI		HR	95% CI	
**All participants (*n*** **= 51,186)**									
Community-level educational attainment^a^	1.04	(1.02–1.07)	*	1.03	(1.01–1.05)	*	1.04	(1.01–1.07)	*
Individual-level educational attainment^b^	1.54	(1.43–1.65)	*	1.11	(1.03–1.19)	*	1.08	(1.01–1.16)	*
Cross-level interaction	0.96	(0.92–0.99)	*	0.98	(0.94–1.01)		0.98	(0.94–1.02)	
**Non-Urban (*n*** **= 20,967)**									
Community-level educational attainment^a^	1.08	(1.03–1.13)	*	1.06	(1.01–1.11)	*	1.07	(1.02–1.13)	*
Individual-level educational attainment^b^	1.67	(1.44–1.93)	*	1.10	(0.95–1.28)		1.07	(0.92–1.25)	
Cross-level interaction	0.92	(0.87–0.98)	*	0.98	(0.91–1.05)		0.98	(0.92–1.05)	
**Urban (*n*** **= 30,219)**									
Community-level educational attainment^a^	1.01	(0.98–1.04)		1.02	(0.99–1.05)		1.03	(0.99–1.06)	
Individual-level educational attainment^b^	1.51	(1.39–1.64)	*	1.11	(1.02–1.21)	*	1.09	(1.00–1.18)	
Cross-level interaction	0.97	(0.91–1.03)		0.98	(0.93–1.04)		0.98	(0.93–1.04)	

*Notes: HR* hazard ratio, *CI* confidence interval; ^a^HR for 10 percentage point increment of proportion of educational attainment in a community area; ^b^HR for educational attainment less than 9 yr. Model 1: crude model + age + sex; Model 2: Model 1 + community-level covariate (population density) + individual-level covariates (income, years of residence, stroke, hypertension, diabetes, hearing loss, frequency of drinking, smoking status, and social isolation). *: *p* < 0.05

In Model 2, among participants living in non-urban areas, community-level high prevalence of low educational attainment showed a statistically significant relationship with dementia risk (HR: 1.07; 95% CI: 1.02–1.13). We found a statistically significant cross-level interaction term (HR: 0.92; 95% CI: 0.87–0.98 in the crude model) such that individuals with low educational attainment showed 8% lower dementia incidence, estimated by 10 percentage points of increment of a proportion of educational attainment in a community area. For the participants living in urban areas, the HRs of community-level high prevalence of low educational attainment were not significant in any of the models (HR: 1.03; 95% CI: 0.99–1.06 in Model 2). In urban areas, individual-level educational attainment showed a significant relationship with a high likelihood of developing dementia (HR: 1.11; 95% CI: 1.02–1.21 in Model 1), but the significance disappeared in Model 2.

## Discussion

This study is the first to reveal contextual associations of community-level prevalence of low educational attainment on developing dementia among older people using longitudinal and large-scale sample data. Older adults living in communities with a higher prevalence of low educational attainment among their age demographic tended to develop dementia more often than those living in areas with a lower prevalence of low educational attainment after adjusting for individual-level educational attainment and covariates. The association was pronounced in non-urban areas.

Our results support the systematic review showing consistent evidence of an association between lower community-level SES indicators, such as the proportion of adult population with no high school degree, and declining cognition after personal SES factors are controlled for. A few longitudinal studies included in the review show that community-level SES was significantly associated with cognitive decline. Social isolation is proposed to be a mediator between low community-level SES and declining cognition [22]. Further, previous studies have also proposed years of residence [7, 22] as a potential confounder. In our models, possible mediators and confounding factors, such as the abovementioned, were fully adjusted to the limitations of previous studies. The current study was a longitudinal study with 50,000 participants, although the systematic review mainly included small cross-sectional studies of fewer than 10,000 participants [22]. Therefore, our study design was more robust than that of prior studies.

We found that the contextual association of community-level prevalence of low educational attainment on dementia incidence. People living in communities with low education among adults might depend much more on community-level rather than individual-level resources due to limitations in residents’ individual-level resources. This means that poor-quality community-level resources can affect community residents’ quality of life and health. Some disadvantages of low community-level SES associated with low education communities (poorly maintained walkways, parks, shopping areas, and neighborhood organizations) were associated with lower cognitive decline [6]. Moreover, previous studies have found that low food store availability and low sidewalk installation is associated with increased dementia incidence [15, 29]. In the current study, this lack of physical and social resources in communities with lower education was associated with dementia incidence compared to communities with higher education.

Bridging social capital, which indicates the connection between different groups or SES levels, can be less developed in communities with low education than in high-education communities because of limited local resources. Therefore, disseminating information and action might be inactive in communities with less education [30]. For example, people living in communities with stronger bridging social capital might quickly acquire health or dementia prevention information through various local networks, an idea called “social contagion” [30, 31]. Moreover, the concept of informal social control shows that people with higher social capital work harder to maintain social order; such individuals ask people with unhealthy lifestyles, who include people with low education, to change their health behaviors. Additionally, people with unhealthy lifestyles can observe correct health behaviors and imitate their actions through community networks [30, 31]. Therefore, improving the bridging of social capital among older adults with lower education could be associated with decreased dementia risk, which is attributable to the associations of social contagion and informal social control.

Conversely, there is strong bonding social capital in communities with high proportion of low educational attainment; people with less education survive and help each other. However, bonding social capital is associated with more psychological disorders [30], which can be associated with dementia risk. Additionally, because communities with less community-level education have more sparse resources than communities with more education, social capital might have some negative impacts, such as placing excessive demands on members. Limited local resources lead to increased pain for a few limited, reliable members of a community and exclusion of newcomers with few years of residence [30]. Generally, because the negative impacts of social capital might be associated with a higher risk of dementia, it is necessary to increase social capital in communities with less educational attainment by developing human and economic resources, such as training community leaders and volunteers; these efforts will build community resources to reduce the negative impacts of social capital [30].

Our study is consistent with review articles that found that people in urban centers tended to develop dementia less than those in rural areas [10, 32]. Studies have found unequal distributions of resources related to better cognitive function (health clinics, bookstores, and libraries) to be associated with lower demands for such resources in less educated communities along with a smaller tax base to pay for the resources [6]. This situation is more pronounced in non-urban areas than in urban areas.

People living in communities with less education, especially non-urban areas, could be disproportionately exposed to chronic and stressful life conditions that generate hazards, such as few employment opportunities and low incomes. This situation results in a lack of social resources (social clubs and neighborhood organizations) and safe physical resources, including gyms [6]. In the current study, the limited resources in non-urban areas compared with those in urban areas could be associated with the increased incidence of dementia.

In one systematic review with a meta-analysis, early life rural living was strongly associated with a risk of Alzheimer’s disease [32]. Additionally, previous studies have found greater educational inequality in rural areas, such as insufficient educational opportunities and poorer quality of education [10, 33]. Although some children left rural areas and continued their studies in urban areas [33], many participants in this study might have long lived in the same rural areas where they had received their education [34]. We did not clarify the quality of education or the childhood residential areas in our study; however, educational disadvantage in rural areas could be associated with greater risk of dementia [34]. Conversely, in a U.S. study, cognitive decline was more significant among adults living in rural areas compared with urban areas in recent decades. This association reflects narrowing rural–urban disparities, which are attributable to increasing education between 1910 and 1940 [9]. The same trend could occur in Japan in the future given that compulsory education was extended from six to nine years in 1947 [21].

This study has some limitations. First, we did not identify transfers of residential areas, including urban to non-urban areas or vice versa. Additionally, we did not identify participants’ childhood residential areas. However, we did adjust for years of residence to overcome the limitations of prior studies [7, 22]. Second, though we did not consider the quality of education, majority of SES indicators in the existing studies were educational years [22]. Third, we lacked data on some potentially community-level confounding factors, such as accessibility of local services, but we considered population density. Fourth, because we did not consider the study survey response rate, there could have been selection bias in our findings. Specifically, because the respondents were healthier than non-respondents, the identified associations might be underestimated. Lastly, we failed to perform a competing risk analysis after conducting multiple imputation with a multilevel model, which could have led to bias related to not considering the competing analysis.

## Conclusions

Older people living in communities with high prevalence of low educational attainment among their age demographic develop dementia more often than those living in areas with lower prevalence of low educational attainment after adjusting for individual-level educational attainment and covariates. Although the association was pronounced in non-urban areas, the identified associations were not strong. These findings establish the foundation for future studies. We do conclude, however, that securing education for adolescents as a life course and population approach might be crucial to preventing dementia later in life among older people living in non-urban areas.

## Supplementary Information

**Additional file 1: Supplementary Table S1**. Criteria of Levels of Cognitive Disability in Japanese Long-Term Care Insurance System. The Ministry of Health, Labor and Welfare in Japan classified eight ranks on a dementia scale according to people’s cognitive disability.

## Data Availability

Data are from the JAGES study. All enquiries are to be addressed at the data management committee via e-mail: dataadmin.ml@jages.net. All JAGES datasets have ethical or legal restrictions for public deposition due to inclusion of sensitive information from the human participants. Following the regulation of local governments that cooperated on our survey, the JAGES data management committee has imposed the restrictions upon the data.

## Abbreviations

CI: Confidence interval
EU: European Union
FUA: Functional Urban Area
HR: Hazard ratio
JAGES: Japan Gerontological Evaluation Study
MHLW: Ministry of Health, Labor and Welfare
OECD: Organisation for Economic Co-operation and Development
SES: Socioeconomic status
