# Correction to: Community-level educational attainment and dementia: a 6-year longitudinal multilevel study in Japan

**DOI:** 10.1186/s12877-021-02668-y

**Published:** 2021-12-15

**Authors:** Tomo Takasugi, Taishi Tsuji, Masamichi Hanazato, Yasuhiro Miyaguni, Toshiyuki Ojima, Katsunori Kondo

**Affiliations:** 1 Department of Community Health and Preventive Medicine, Hamamatsu University School of Medicine, 1-20-1 Handayama, Higashi-ku, Hamamatsu, Shizuoka, 431-3192 Japan; 2 Faculty of Health and Sport Sciences, University of Tsukuba, 3-29-1 Otsuka, Bunkyo-Ward, Tokyo, 112-0012 Japan; 3 Center for Preventive Medical Sciences, Chiba University, 1-33 Yayoi-cho, Inage-Ward, Chiba, 263-8522 Japan; 4 Design Research Institute, Chiba University, 1-19-1 Bunka, Sumida-ku, Tokyo, 131-0044 Japan; 5 Faculty of Social Welfare, Nihon Fukushi University, Okuda, Mihama-cho, Chita-gun, Aichi 470-3295 Japan; 6 Center for Gerontology and Social Science, National Center for Geriatrics and Gerontology, 7-430 Morioka-cho, Obu-City, Aichi 474-8511 Japan

**Correction to: BMC Geriatr 21, 661 (2021)**

**https://doi.org/10.1186/s12877-021-02615-x**

After publication of this article [1], the authors reported that the ORCID IDs for all authors were missing, as well as the second affiliation of Masamichi Hanazato. Moreover, some other, small errors were corrected.

The original article [1] has been updated.
