# Supplementary material for: Community-level educational attainment and dementia: a 6-year longitudinal multilevel study in Japan
Source: BMC Geriatr. 2021 Nov 23;21:661. doi: 10.1186/s12877-021-02615-x (PMC8609807; doi:10.1186/s12877-021-02615-x)
Supplement: Supplementary file 1 — Additional file 1: Supplementary Table S1. Criteria of Levels of Cognitive Disability in Japanese Long-Term Care Insurance System. The Ministry of Health, Labor and Welfare in Japan classified eight ranks on a dementia scale according to people’s cognitive disability. [file 12877_2021_2615_MOESM1_ESM.docx]

**Supplementary Table S1. Criteria of Levels of Cognitive Disability in Japanese Long-Term Care Insurance System**

| Rank | | Criteria | Examples of observable symptoms or behaviors |
| --- | --- | --- | --- |
| Independent | |  |  |
| I | | Suffers from a certain dementia symptoms, but the daily living is almost all independent in the domestic and social spheres. |  |
| II | | Manifests some symptoms/behaviors and communication difficulties that may hinder the daily activities, but can be independent if someone takes care them. |  |
|  | IIa | The abovementioned conditions in II are observed while outside the domestic sphere. | Frequently gets lost on the street, or makes noticeable mistakes in matters that the person was previously able to handle, such as shopping, personal administrative tasks, or financial management. |
|  | IIb | The abovementioned conditions in II are also observed in the domestic sphere. | Is unable to manage taking medication or stay alone at home due to an inability to answer the phone or the door. |
| III | | Occasionally manifests communication difficulties or symptoms/behaviors that hinder daily activities, thus requiring care. |  |
|  | IIIa | Manifests above mentioned conditions described in III predominantly during the day. | Has difficulty or takes time to change clothes, take meals, defecate, or urinate; puts objects into the mouth, picks up and collects objects, is incontinent, makes loud and incoherent screams, carelessly handles fire, or engages in unhygienic acts or inappropriate sexual acts, etc. |
|  | IIIb | Manifests abovementioned conditions described in III predominantly at night. | Same as rank IIIa. |
| IV | | Frequently manifests difficulties communicating or symptoms/behaviors that hinder daily activities and constantly requires care. | Same as rank III. |
| M | | Manifests significant mental symptoms, problematic behaviors, or severe physical illnesses and requires specialised medical care. | Shows continued mental symptoms, such as delirium, delusions, and agitation, and manifests associated problematic behaviors, such as self-mutilation or harm to others. |

Ministry of Health, Labour and Welfare. Textbook for expert investigators concerning a certification of needed long-term care, revised edition 2009. Tokyo, Japan: Ministry if Health, Labour and Welfare. [www.mhlw.go.jp/file/06-Seisakujouhou-12300000-Roukenkyoku/0000077237.pdf](http://www.mhlw.go.jp/file/06-Seisakujouhou-12300000-Roukenkyoku/0000077237.pdf). [in Japanese] Published 2018. Accessed February 2, 2021.
